# Predictive Value of Credit Score on Surgery Resident and Fellow Academic and Professional Performance

**DOI:** 10.7759/cureus.15946

**Published:** 2021-06-26

**Authors:** James A Berry, Dario A Marotta, Paras Savla, Emilio C Tayag, Saman Farr, Rida Javaid, Daniel K Berry, Sara E Buckley, Anna Rogalska, Dan E Miulli

**Affiliations:** 1 Neurosurgery, Riverside University Health System Medical Center, Moreno Valley, USA; 2 Research, Alabama College of Osteopathic Medicine, Dothan, USA; 3 Neurology, University of Alabama at Birmingham, Birmingham, USA; 4 Neurology and Neurosurgery, Desert Regional Medical Center, Palm Springs, USA; 5 Medicine, Donald and Barbara Zucker School of Medicine at Hofstra/Northwell, Hempstead, USA; 6 Medicine, Peconic Bay Medical Center-Northwell Health, Riverhead, USA; 7 Flight Surgery, Federal Aviation Adminstration, Kansas City, USA; 8 Orthopedics, University of the Incarnate Word School of Osteopathic Medicine, San Antonio, USA; 9 General Surgery, University of Texas Health Science Center, San Antonio, USA; 10 Neurosurgery, Arrowhead Regional Medical Center, Colton, USA

**Keywords:** credit score, surgery, residency, performance, graduate medical education

## Abstract

Introduction

Surgical specialties consistently remain among the most competitive residency and fellowship programs with some of the highest rates of unmatched applicants. Attrition in surgical specialties is as high as 30% and particularly problematic given the extended duration of training and limited number of positions. Applicants are traditionally evaluated using a streamlined set of objective metrics, such as board scores, class rank, leadership, letters of recommendation, research productivity, and volunteerism. Consumer credit scores have been shown to be predictors of personality and work performance, however, the literature has yet to explore consumer credit histories in the context of surgical resident and fellow performance. This study aims to determine whether consumer credit scores of surgery residents and fellows are predictive of academic and professional performance.

Methods

This is a multi-institutional observational survey study across all American Council of Graduate Medical Education and Royal College of Physicians and Surgeons accredited surgical residency and fellowship programs in the United States and Canada. Ninety-nine surgical residents and fellows with educational status of post-graduate year two or higher participated in this study. Dichotomous (yes or no) survey items were formulated to assess performance indicators in the domains of notable achievements and awards, research output, written examination performance, professionalism, and surgical/technical skills. Three-digit Fair Isaac Corporation (FICO) credit scores, a widely accepted consumer reporting score, were collected to avoid calculation variability between algorithms.

Results

Surgical residents and fellows reported credit scores between 611( fair) and 853 (exceptional) with a median (interquartile range) of 774 (715-833). The majority of participants 51.5%(51) reported very good credit scores. Those with higher credit scores (very good/exceptional) were 377% more likely to have one or more positive performance indicators OR (95% CI) = 3.77 (1.43-9.97). Similarly, residents with lower credit scores (fair/good) were only 40% more likely to have one or more negative performance indicators. The credit score has a moderate ability to distinguish between the presence and absence of positive performance indicators (area under the curve {AUC} = 0.70, p = 0.001). The use of 753 as a credit score cutoff is 78.9% sensitive and 52.4% specific for discerning surgery residents and fellows with one or more positive performance indicators. The credit score did not significantly discern those with negative performance indicators.

Conclusions

While credit score was significantly functional in discerning those with and without positive performance indicators, sensitivity and specificity rates leave much to be desired. Although our data may have indicated that higher credit scores may be associated with increased residency academic performance on examinations and research productivity we are not recommending any implementation of using credit scores as a metric for selecting individual surgical candidates for a position in residency or fellowship due to extensive socioeconomic variables and historical context of credit scores, which must be taken into consideration. Additional studies are needed to assess this utility on a larger scale.

## Introduction

Graduating medical students and senior residents vie for a limited number of residency and fellowship programs every year in the United States and Canada. These programs vary in size, often consisting of between one and 220 residents and fellows. Program size and years of training change by specialty and program type. For example, surgical specialties offer fewer overall positions and have extended lengths (five to seven years) of training compared to non-surgical programs, which have more positions and shorter durations (one to five years). In the United States, only 20% of the 140,000 residency and fellowship positions available through the National Resident Match Program were surgically related. As such, surgical specialties consistently remain among the most competitive programs with some of the highest rates of unmatched applicants [1].

Given the popularity of surgical specialties and the high number of competitive applicants, program directors are tasked with an annual dilemma: selecting residents and fellows from a set of uniquely qualified individuals. The applicants are traditionally evaluated using a streamlined set of objective metrics such as board scores, class rank, leadership, letters of recommendation, research productivity, and volunteerism. A pragmatic approach to resident and fellow selection melds these objective measures with subjective impressions gleaned through direct candidate interaction during elective rotations and interviews. Nevertheless, the resident and fellowship selection process remain imperfect and fallible. While the majority of positions at competitive surgery programs are filled each year, attrition in surgical specialties is particularly problematic with rates as high as 30% [2]. Uncontrollable lifestyle, lack of support, and length of training were all cited as factors for attrition; none of which have been shown to be predicted by the above-mentioned objective and subjective measures [3,4]. This prompts the need for further investigation into new assessment measures to predict the success, performance, and longevity of future residents and fellows.

Historically, consumer credit scores have been used by financial lenders to assess financial capacity, namely the ability to accept and repay debt. On the surface, credit scores provide a three-digit numeric value using mathematical algorithms with data from an applicant’s financial history. Many scoring models exist. Depending on the scoring model used, weights are applied to several factors, such as the number of credit accounts, types of accounts, ages of accounts, debt versus available credit ratio, and payment history. The scores often range from 300 (bad) to over 850 (excellent). Over time, credit score was found to be a predictor of candidate personality and job performance in the workplace [5,6]. Studies emerged evaluating credit scores as surrogates for performance, extraversion, agreeableness, openness, conscientiousness, and neuroticism [7,8]. However, the literature lacks a detailed characterization of consumer credit score in the setting of graduate medical education. Therefore, we set out to evaluate the utility of credit score as a predictor of positive and negative performance indicators in surgical residents and fellows.

## Materials and methods

This is a multi-institutional observational survey study across all American Council of Graduate Medical Education and Royal College of Physicians and Surgeons accredited surgical residency and fellowship programs in the United States and Canada. Surgical residents and fellows with educational status of post-graduate year two or higher were invited to take part in this study. Contact information for 1089 program coordinators of general surgery, integrated plastic surgery, integrated vascular surgery, neurosurgery, orthopedic surgery, otolaryngology, and urology programs were compiled using the database from the American College of Surgeons, American College of Osteopathic Surgeons, American Association of Neurological Surgeons and other professional societies. Program coordinators were sent electronic requests soliciting resident study participation, 50 of which returned undeliverable. Electronic communications included instructions and a hyperlink to complete the online survey. This study was approved as exempt status by the Arrowhead Regional Medical Center Institutional Review Board. Individual consent was implied through survey completion.

Survey

The survey consisted of an online 10-question survey link that was emailed to the individual program coordinators. A list of the survey questions by performance indicator type (positive vs. negative) is displayed in Table 1. The information contained within the survey was inherently private and sensitive. Thus, the survey was designed to be completed without gathering any of the participant's personal identification information and demography (i.e., name, social security number, address, age, sex, and residency/fellowship program name) to minimize the risk of financial or educational information disclosure and maximize participation. Dichotomous (yes or no) survey items were formulated to assess performance indicators in the domains of notable achievements and awards, research output, written examination performance, professionalism, and surgical/technical skills. One item served to identify participants with histories of financial hardship by means of a disadvantaged background (i.e., public aid, food stamps, welfare, etc.). Three-digit Fair Isaac Corporation (FICO) credit scores, a widely accepted consumer reporting score, were collected to avoid calculation variability between algorithms. 

**Table 1:** List of survey questions and performance indicator types FICO = Fair Isaac Corporation; ABSITE = American Board of Surgery In-Training Examination; PGY = post-graduate year

No.	Indicator Type	Question Text
1	-	What is your three-digit Fair Isaac Corporation (FICO) credit score?
2	Positive	Have you ever received an award from your residency program such as a Resident of the Year Award or received an Award for a Publication you were primary author on?
3	Negative	Have you ever been placed on probation for an academic reason?
4	Positive	Have you ever scored at the top of your program on an annual in-service examination (i.e., ABSITE)?
5	Positive	Have you averaged publishing at least one peer-reviewed journal article or textbook chapter per academic year in your residency?
6	Negative	Have you ever been placed on probation for a professional or behavioral reason?
7	Negative	Have you ever scored below average for your PGY level on your annual in-service examination (i.e., ABSITE)?
8	Negative	Have you ever had to repeat a rotation or academic year for a non-medical/non-personal reason?
9	Negative	Have you recently been told that your surgical/technical skill is below average for your current academic PGY level?
10	-	Do you come from a financially disadvantaged background (i.e., public aid, food stamps, welfare, etc.) which may interfere with your credit score?

Procedure

The survey items were sorted by positive and negative performance indicators. Three positive performance indicators measured a participant’s history of achievements awarded during residency, research output, and top in-service examination scores. Five negative performance indicators measured history of below average in-service examination scores, academic probation, probation for professionalism or behavioral violations, repeat residency rotations, and below average surgical or technical skills. Positive and negative scores were tallied by participants. A net performance value was calculated by subtracting the cumulative positive performance indicators from negative performance indicators. Participant performance was classified as the net negative, net neutral, and net positive according to negative, neutral (zero), and positive net performance values, respectively. The credit scores were sorted by value into a categorical variable based on poor (<580), fair (580-669), good (670-739), very good (740-799), and exceptional (+800). Additionally, categories were grouped into high (exceptional and very good) and low (good, fair) credit score groups.

Data analyses

Chi-squared tests were utilized to compare the proportion of surgery residents with positive and negative performance indicators across high (exceptional, very good) and low (good, fair) credit score groups. Results were interpreted as OR where values of 2.0, 3.0, and 4.0 correspond to minimum, moderate, and strong risk estimates, respectively [9]. Here, we used the phi (φ) coefficient to interpret effect size (φ = [𝜒^2^/N]^1/2^), where values of 0.2, 0.5, and 0.8 correspond to minimal, moderate, and strong effect sizes, respectively [10].

Kolmogorov-Smirnov and Shapiro-Wilk tests were used to assess normality for credit score continuous data; analyses revealed evidence of non-normality of the distribution of credit scores (D{99} = 0.13 , p < 0.001; W{99} = 0.92, p < 0.001) [11]. As such, nonparametric tests were used for between-group comparisons. We first compared differences in credit scores across the performance indicator groups (i.e., net negative, neutral, and positive) using a Kruskal-Wallis H test [12]. We used the eta^2^ (η^2^) coefficient and an index of Kruskal-Wallis H effect size (η^2^ = 𝜒^2^ / (n-1)) with 0.4, 0.25, 0.64 correspondings to minimal, moderate, and strong effect sizes, respectively [10]. We treated the Kruskal-Wallis H test analysis as an omnibus test, where a significant overall effect was then followed up by pair-wise post hoc Mann-Whitley U tests to determine pair-wise differences among the groups [13]. We used the r coefficient to index Mann-Whitley U effect sizes (r = z / (n)^1/2^), which were interpreted using 0.2, 0.5, and 0.8 as cutoffs for minimal, moderate, and strong effect sizes, respectively [10].

Lastly, receiver operating characteristic curves (ROC) analyses were used to assess the practical utility of credit score in the selection of surgery residents/fellows to discern performance indicators. These analyses were interpreted using the area under the curve (AUC) values of < 0.70 indicating low classification utility, AUC values between 0.70 and 0.90 indicating moderate classification utility, and AUC over 0.90 indicating high classification utility. The data were analyzed in SPSS software version 27 (Armonk, NY: IBM Corp.).

## Results

Ninety-nine surgery residents participated in the study. Table 2 displays the frequency and descriptive measures of survey results.

**Table 2:** Descriptive statistics and frequencies of survey responses ^a^Reported as median (IQR). IQR = interquartile range; FICO = Fair Isaac Corporation

Variable	n (%)
Credit Score (FICO)	774 (59)^a^
FICO Categories:	
Poor (<580)	0 (0.0%)
Fair (580-669)	4 (4.0%)
Good (670-739)	20 (20.2%)
Very Good (740-799)	51 (51.5%)
Exceptional (800+)	24 (24.2%)
Positive Performance Indicators	
Awards	19 (19.2%)
Research Publications (1 per year)	41 (41.4%)
Top Exam Performer	19 (19.2%)
Negative Performance Indicators	
Academic Probation	0 (0.0%)
Behavioral/Professional Probation	4 (4.0%)
Below Average Exam Performer	29 (29.3%)
Below Average Surgical Skills	2 (2.0%)
Repeat Rotation	1 (1.0%)

Credit scores

Participants reported credit scores between 611 and 853 with a median (interquartile range) of 774 (715-833). The majority of participants, 51 (51.5%), reported very good credit scores. Exceptional and good credit scores were reported by 24 (24.2%) and 20 (20.2%) of respondents, respectively. None of the participants reported poor credit history.

Performance indicator survey results

**Figure 1:**
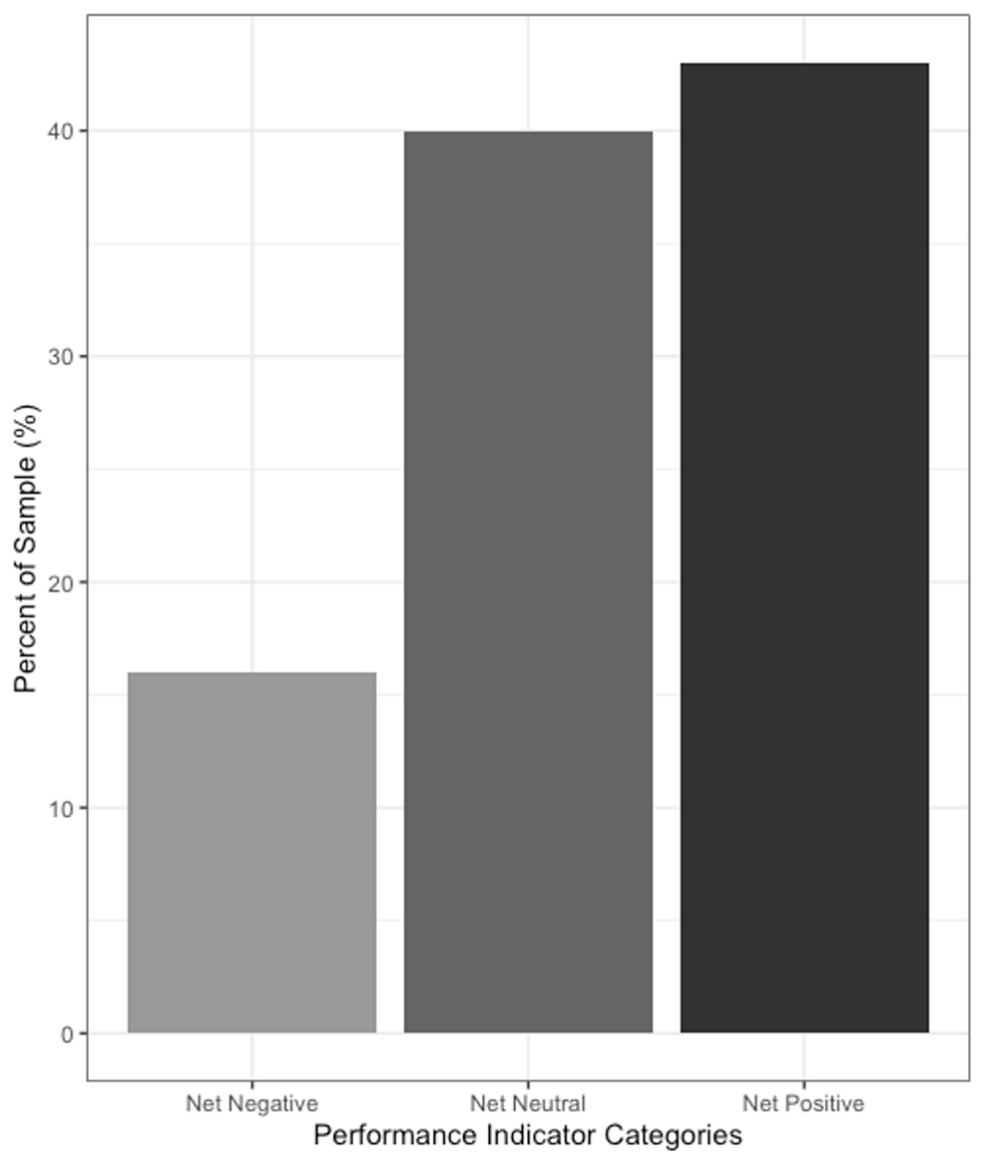
Percent of study participants with positive, negative, and net performance indicators

Among the positive performance indicators, 41 (41.4%) of the participants reported an average of one research publication per year, while only 19 (19.2%) reported an achievement award or top examination score within his or her residency program. An academic achievement award includes categories such as an individual programs resident of the year award, state and national achievement awards for research publications, etc. In general, positive performance indicators were reported more frequently than negative performance indicators. Twenty-nine (29.3%) of the respondents reported below-average examination scores on a national residency examination while none of the participants reported a history of academic probation. A modicum of participants reported prior behavioral or professional probation, 4 (4.0%), residency evaluations documenting below average surgery skills, 2 (2.0%), or having to repeat a residency rotation, 1 (1.0%). Figure 1 below displays the frequency of participants with net negative, neutral, and positive performance values. The majority of participants had a total of zero cumulative positive indicators, zero cumulative negative indicators, and zero net performance indicators. Only 2% of participants had either three cumulative positive indicators, two negative net indicators, or three positive net indicators each.

Quantitative analyses

Comparisons between those with high credit scores (exceptional, very good) and low credit scores (good, fair) by positive and negative performance predictors are displayed in Table 3. Chi-squared analyses revealed a greater proportion of residents with one or more positive performance indicator in those with high credit scores compared to those with low credit scores, 65.3% vs. 33.3%; χ^2^ (1, n = 57) = 7.62, p = 0.006, φ = 0.37. The analysis also indicated that residents with high credit scores had 377% higher odds of having one or more positive performance indicators, OR (95% CI) = 3.77 (1.43-9.97). In contrast, significant differences in the number of negative performance indicators between those with low and high credit scores was not observed, 41.7% vs. 30.7%; χ^2^ (1, n = 33) = 0.99, p = 0.33 φ = 0.17.

**Table 3:** Comparisons of credit score by positive and negative performance indicators ^a^High credit scores are classified as FICO categories of exceptional (800+) and very good (740-799). ^b^Low credit scores are classified as FICO categories of good (670-739) and fair (580-669). FICO = Fair Isaac Corporation

Variable	High Credit Score^a^ n (%)	Low Credit Score^b^ n (%)	χ^2^ (p)	OR (95% CI)
Positive Performance Indicators				
1 or more positive indicators	49 (65.3%)	8 (33.4%)	7.62 (.006)	3.77 (1.43-9.97)
No positive indicators	26 (34.7%)	16 (66.7%)		
Negative Performance Indicators				
1 or more negative indicators	23 (30.7%)	10 (41.7%)	0.99 (0.331)	0.62 (0.24-1.60)
No negative indicators	52 (69.3%)	14 (58.3%)		

Kruskal-Wallis test showed that significant differences among credit scores of surgical residents/fellows with net negative, net neutral, and net positive performance indicators exist, H(2) = 7.49, p = .024, η^2^ = 0.076). Median (interquartile range) credit scores were found to be 747 (65), 756 (49), and 774 (40) for those with net negative, neutral, and positive performance indicators, respectively. Post-hoc Mann-Whitney tests were used to compare all pairs of groups. Median credit scores of surgical residents/fellows with net negative or net neutral performance indicators were significantly lower than those with net positive indicators, U = 844, p = 0.11, r = 0.26. Similarly, the difference between credit scores of residents/fellows with no positive indicators compared to those with positive indicators was also significantly lower, U = 718, p = 0.001, r = 0.34). In contrast, credit scores were lower for those with negative performance indicators compared to those without, although differences were no significant, U = 984, p = 0.436, r = 0.078).

Practical utility

**Figure 2:**
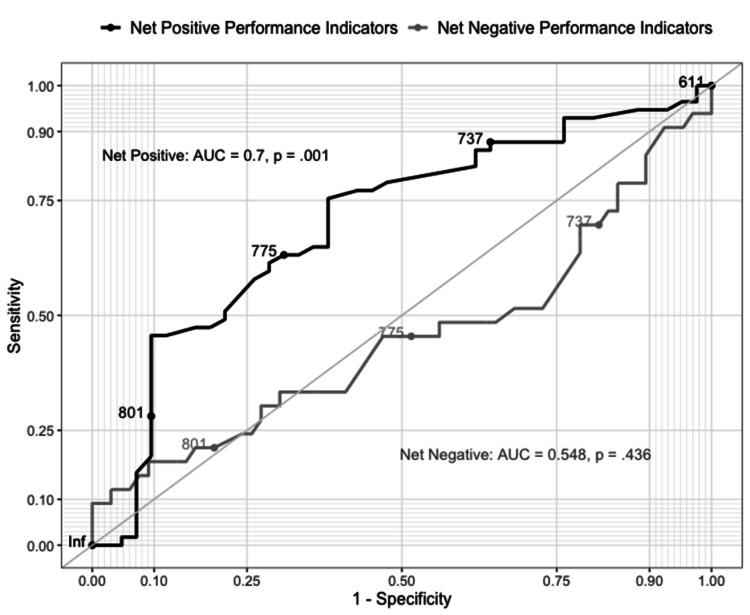
Receiver operator curves for the utility of credit score to discern positive and negative resident performance AUC = area under the curve

To assess the credit score’s ability to discern resident/fellow performance, credit scores were subjected to AUC analysis of ROC curves (Figure 2). Credit score has a moderate ability to distinguish between the presence and absence of positive performance indicators (AUC = 0.70, p = 0.001). The optimal cutoff for this ROC analysis was a credit score of 753, with values above or equal this being 78.9% sensitive and 52.4% specific for discerning surgical residents with one or more positive performance indicators. In contrast, ROC analysis indicated that credit score did not significantly detect negative performance, and as such classification accuracy rates were poor (AUC = 0.548, p = 0.436).

## Discussion

We used a survey-based multi-institutional approach to evaluate the utility of credit scores as a predictor of positive and negative performance indicators. Overall, we found that surgical residents and fellows had fair to exceptional credit scores. Surgical residents and fellows with high credit scores (exceptional, very good) were much more likely to have positive performance indicators, such as top written examination performance, multiple research publications, and professional awards and recognition. We found that the use of 753 as a credit score cutoff is 78.9% sensitive and 52.4% specific for discerning surgery residents with one or more positive performance indicators. However, credit scores did not significantly discern those with negative performance indicators. We found that low credit scores (good, fair) did not predict negative performance indicators such as below-average performance (written examinations and surgical skills), probation status (i.e., academic, behavioral, professional), and repeated rotations. To our knowledge, this is the first study in the literature to investigate credit history in the context of graduate medical education.

The utility of credit scores as a potential screening method relies on its potential function as a construct of historical decision-making. That is, the idea that credit scores represent habits, character traits, and decision-making leading to financial stability, rather than socioeconomic status alone. Generally, negative factors (i.e., short duration of credit history, many credit inquiries, high debt to credit ratios, and late payments) reduce credit score, while the opposite trends increase credit score. Although each factor is related to fiscal responsibility, they also can serve as surrogates of behaviors and habits. Late payments on outstanding balances may suggest poor organization, irresponsibility, and lack of follow-through. High credit card balances secondary to overspending may reveal a lack of self-discipline and restraint. Numerous credit inquiries and outstanding debts may suggest poor planning for the future. Considering these scores can date back as far as ten years, credit scores can give rise to a unique assessment of a candidate which is not currently available.

In this study, we found higher credit scores significantly predicted those with top written examination performance, multiple research publications, and professional awards and recognition. This is consistent with past literature showing that higher credit scores translate to higher work-related performance [7]. Taking part in extracurricular activities, such as research, and studying enough to score highly on written examinations during the rigors of post-graduate education takes persistent motivation, commitment, and emotional stability. Similar discipline is necessary to achieve and maintain high credit scores. It is also conceivable that those who commit to success in one domain (i.e., creditworthiness) have the innate drive to remain consistent in other domains of life (i.e., positive performance during residency) [14,15]. Thus, in a functional sense, credit scores may have the ability to detect candidates who are simply driven to succeed.

In contrast, lower credit scores did not predict below-average performance in academic and surgical skills, probationary status, or repeated clinical rotations. This may arise from the positive skew of credit scores represented by those within surgery-related programs. The paucity of lower credit scores in this cohort (i.e., poor, fair) may prevent such a prediction. That is, credit scores included in the cohort are not truly “lower” credit scores required for such an association. Moreover, this study surveyed residents and fellows who have already attained a position in residency and fellowship, and those with negative credit scores may have already been eliminated or succumbed to attrition [16]. Nevertheless, those who attain graduate medical education in surgical specialties share many personality traits including extroversion, conscientiousness, and emotional intelligence; all of which have been associated with positive credit scores [7,17,18]. Those who are extroverted tend to be increasingly energetic, can effectively network, and engage in interpersonal activities such as collaborative research, while those who are conscientious may be more reliable and self-controlled with a focus on planning, organizing, and satisfying responsibilities [7,8]. Finally, those with a high degree of emotional intelligence are less likely to succumb to burnout [19,20]. Taken together, the narrow range of relatively high credit scores of surgery trainees who successfully matched may explain the lack of significance for predicting low performance with lower credit history.

Current metrics for assessing medical residents and fellows are aimed at predicting candidate success during residency. While the framework by which individual surgery programs define success varies, many attributes are widely accepted and emphasized as important. We found that credit scores above 753 had a 79% sensitivity in discerning candidates with one or more positive performance indicators, however, specificity was lacking at 52%. On a practical level, the utility of credit score may function better as a supplement rather than a stand-alone metric in predicting future success to avoid issues with false negatives. Nevertheless, it is important to point out that credit score is subject to influence by socioeconomic status, unforeseen life events, and even fraud. As such, ethical and legal controversies exist when using this score as an indicator of merit [21]. In these instances, it is unclear how the predictive value of credit score holds up. Careful consideration of life events, particularly those which negatively impact a credit score, would likely need to be considered independently.

Limitations and future studies

This study is not without limitations. To start, sociodemographic, socioeconomic, and professional variables were not collected in this study to encourage participation. Surgery programs are relatively small (sometimes training one resident per year) and the potential risk of identifying a study participant or exposing a participant’s credit score may deter individuals from participating. Future studies should include these variables for additional comparisons and to assess for potential confounding variables. Secondly, this study relies on self-reported credit scores. While self-reported credit scores have been shown to be a reliable measure of actual credit scores, it is possible that some scores were not accurately or honestly reported [22]. Next, the cross-sectional method of the study reveals results from a single time point. Credit scores are constantly in flux and a longitudinal study may be useful to determine associations between changes in credit scores and changes in performance for residents and fellows as they progress throughout their careers. Finally, the inclusion of residents and fellows who succumbed to attrition may also help determine if financial strain may have been a factor in dropping out.

## Conclusions

Overall, we found that surgical residents and fellows in our study had fair to exceptional credit scores. Surgical residents and fellows with high credit scores (exceptional, very good) were 377% more likely to have one or more positive performance indicators. Similarly, residents with low credit scores (good, fair) were only 40% more likely to have one or more negative performance indicators. While credit score is significantly functional in discerning between those with and without positive performance indicators, sensitivity and specificity rates must be considered. The use of 753 as a credit score cutoff is 78.9% sensitive and 52.4% specific for discerning surgery residents with one or more positive performance indicators. Credit scores did not significantly discern those with negative performance indicators. Although our data may have indicated that higher credit scores may be associated with increased residency academic performance on examinations and research productivity we are not recommending any implementation of using credit scores as a metric for selecting individual surgical candidates for a position in residency or fellowship due to extensive socioeconomic variables and historical context of credit scores, which must be taken into consideration. However, additional data and research is needed to fully explore the use of credit score in additional domains such as probationary status, surgical skill, and professionalism.
