# Correction: Predictive Value of Credit Score on Surgery Resident and Fellow Academic and Professional Performance 

**DOI:** 10.7759/cureus.c55

**Published:** 2021-12-13

**Authors:** James A Berry, Dario A Marotta, Paras Savla, Emilio C Tayag, Saman Farr, Rida Javaid, Daniel K Berry, Sara E Buckley, Anna Rogalska, Dan E Miulli

**Affiliations:** 1 Neurosurgery, Riverside University Health System Medical Center, Moreno Valley, USA; 2 Research, Alabama College of Osteopathic Medicine, Dothan, USA; 3 Neurology, University of Alabama at Birmingham, Birmingham, USA; 4 Neurology and Neurosurgery, Desert Regional Medical Center, Palm Springs, USA; 5 Medicine, Donald and Barbara Zucker School of Medicine at Hofstra/Northwell, Hempstead, USA; 6 Medicine, Peconic Bay Medical Center-Northwell Health, Riverhead, USA; 7 Flight Surgery, Federal Aviation Adminstration, Kansas City, USA; 8 Orthopedics, University of the Incarnate Word School of Osteopathic Medicine, San Antonio, USA; 9 General Surgery, University of Texas Health Science Center, San Antonio, USA; 10 Neurosurgery, Arrowhead Regional Medical Center, Colton, USA

This article has been corrected per the request of the authors. Two sentences (one in the abstract and one in the conclusions) have been corrected as detailed in the authors' comments below:

In good faith, we write to report an error and intellectual blind spot in our article that needs to be corrected and clarified. The abstract and conclusion contain the statements: 1) "This study suggests credit score may have a utility as a companion to traditional metrics used in identifying candidates for surgery residencies and fellowships who will have positive performance in the domains of research productivity, written examination performance, and professional awards and recognition”; and 2) “This study suggests credit score may be a valuable tool in identifying candidates for surgery residencies and fellowships who will have positive performance in the domains of research productivity and examination performance.” These statements are not supported by the data contained within the study. As discussed below, these sentences from the former abstract and conclusion should more appropriately read,

“Although our data may have indicated that higher credit scores may be associated with increased residency academic performance on examinations and research productivity we are not recommending any implementation of using credit scores as a metric for selecting individual surgical candidates for a position in residency or fellowship due to extensive socioeconomic variables and historical context of credit scores, which must be taken into consideration.”

While credit scores have been applied as a construct of historical decision-making in certain instances, it is undeniable that sociodemographics, socioeconomics, and life events confound substantive interpretations and preclude practical utility as a metric of suitability, as we mentioned in the paper. The paper reports the survey of residents and fellows currently in surgical programs and their reported credit score and their reported performance. There was no survey of candidates applying for residency or fellowship programs. There were no reported credit scores below 611, therefore there were no poor credit scores.

However, when considering credit score as a relative gauge of financial well-being in the context of this study, the data more appropriately shed light on the impact of financial well-being on resident and fellow performance. Furthermore, we feel it is necessary to reiterate the data do not support the use of credit score for identification or stratification of surgical residents and fellows. In fact, we posit that the use of such a metric for applicant suitability has discriminatory implications that could serve as a barrier for individual residents and undermine racial and class representation within surgery, which in turn could negatively impact patient care. Any additional research on this topic should focus on providing financial support and resources to those who may be at risk for financial well-being as described by the data.

We thank the individuals who participated in the commentary and assessment of this publication. We apologize for interpreting the data in isolation of social realities and drawing a conclusion based on an incomplete perspective. We value this criticism and will use it as an opportunity to better assess our own biases and those within the world around us.

